# Supplementary figures and images for: Systematic Localization of Escherichia coli Membrane Proteins
Source: mSystems. 2020 Mar 3;5(2):e00808-19. doi: 10.1128/mSystems.00808-19 (PMC7055658; doi:10.1128/mSystems.00808-19)

Figure S1

A

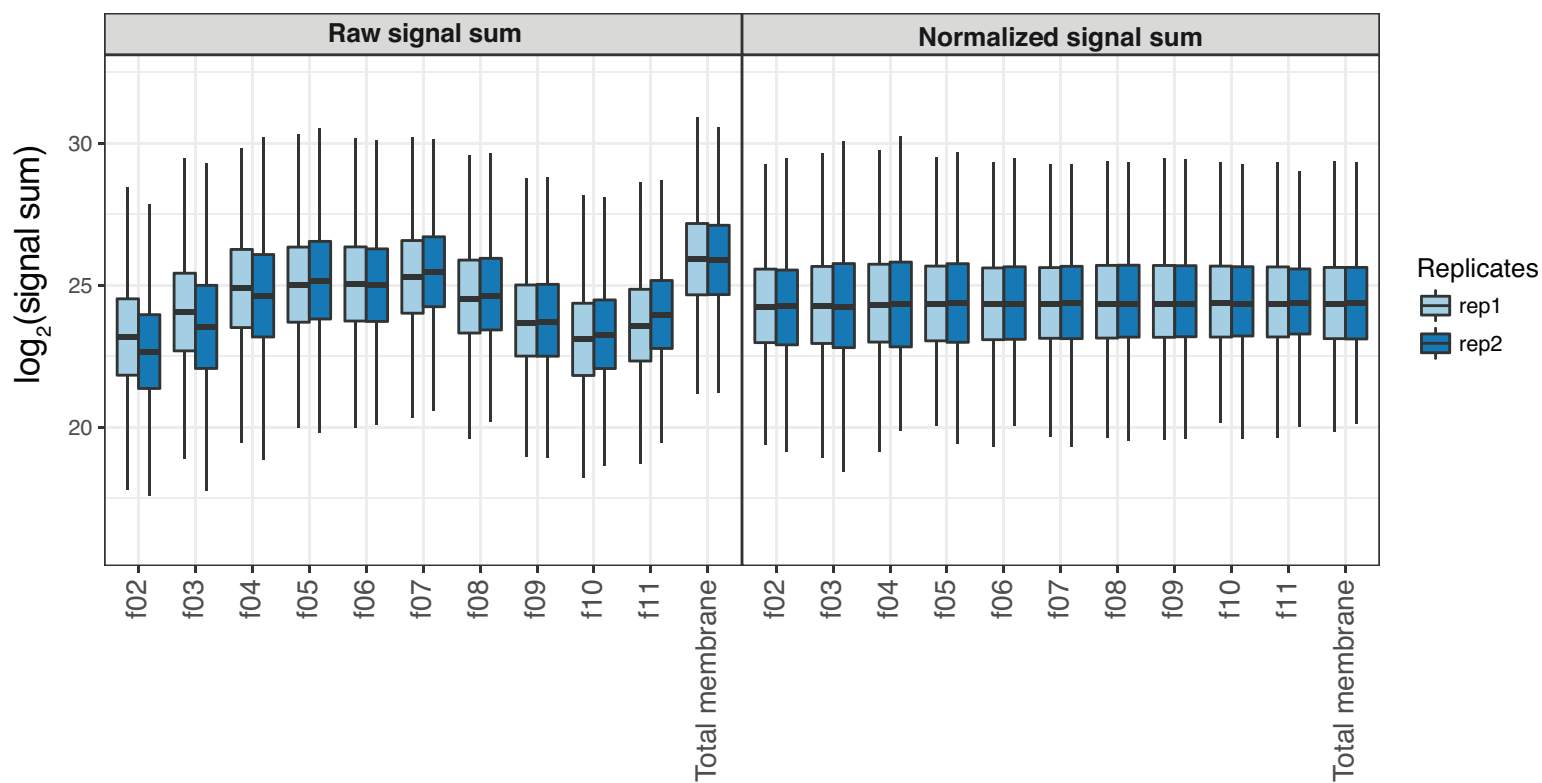

B

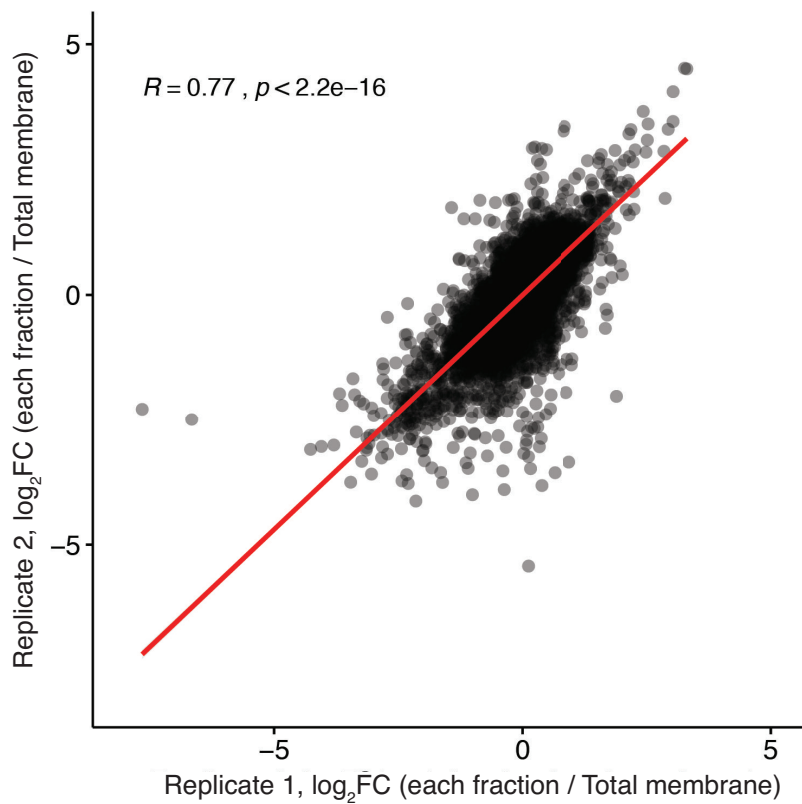

Supplement: FIG S1 [file mSystems.00808-19-sf001.pdf]

Figure S2

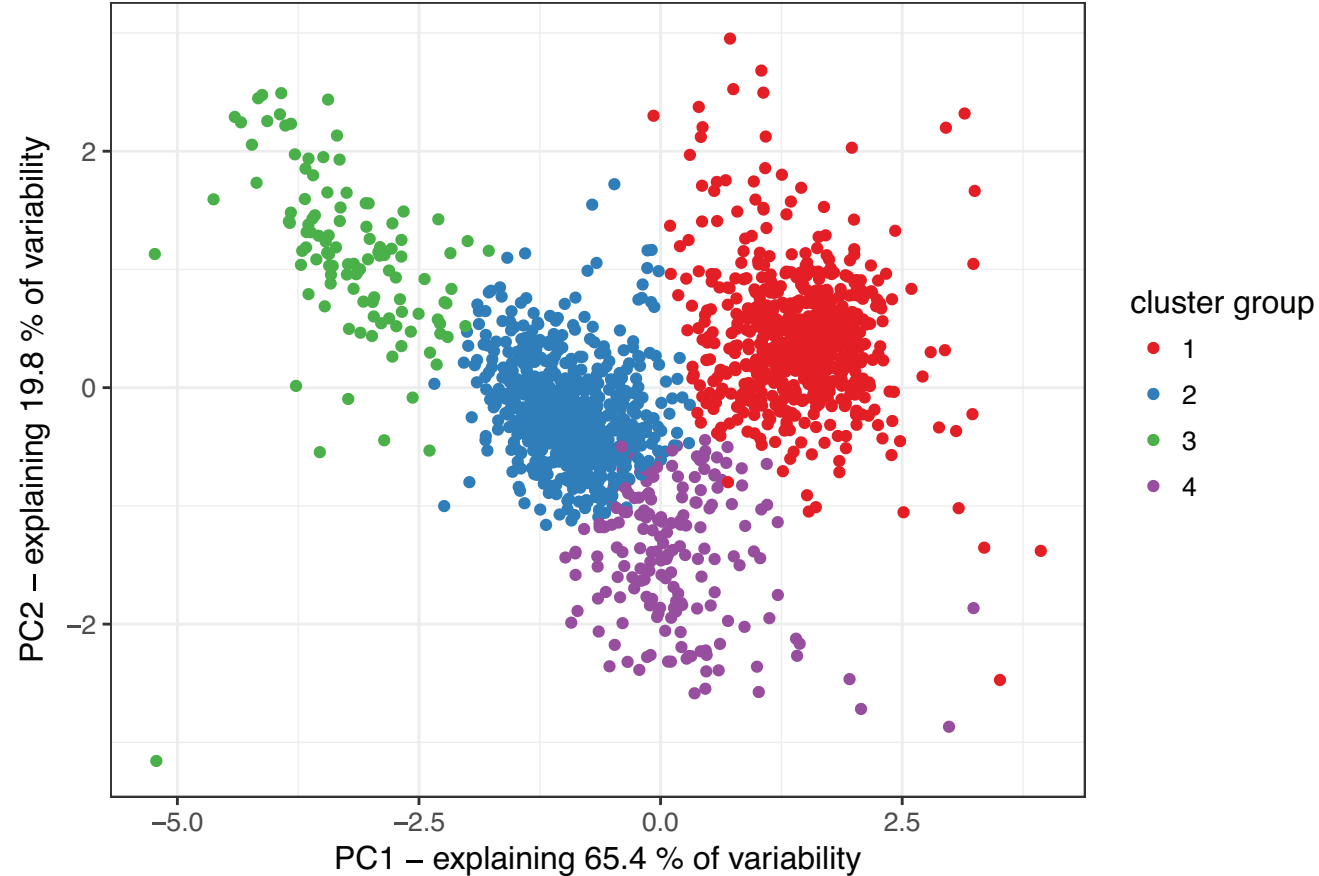

Supplement: FIG S2 [file mSystems.00808-19-sf002.pdf]
